# Effect of Mechanical Strain on Cells Involved in Fracture Healing

**DOI:** 10.1111/os.12885

**Published:** 2021-01-25

**Authors:** Zheng‐wei Duan, Hua Lu

**Affiliations:** ^1^ Department of Orthopaedics Xinhua Hospital Affiliated to Shanghai Jiao Tong University School of Medicine Shanghai China

**Keywords:** Biomechanics, Fracture healing, Mesenchymal stem cell, Osteoblast, Osteoclast

## Abstract

Secondary fracture healing is a complex multi‐stage process in which the mechanical environment plays a key role. The use of an appropriate mechanical stimulation such as strain is conducive to tissue formation between fracture ends, thus aiding the healing process. However, if the strain is too large or too small, the biological behavior of the cells involved in bone healing will be affected, resulting in non‐union or delayed healing. In this review, we summarize the current state of knowledge regarding the effect of strain on cells that play a role in the fracture‐healing process. Overall, the related literature suggests that selection of an adequate strain promotes fracture healing through the stimulation of angiogenesis and osteogenesis, along with inhibition of osteoclast differentiation and bone resorption. However, standardized methods for the application of mechanical stimulation are lacking, and a unified consensus on the mechanism by which strain promotes cell differentiation has not yet been reached. These issues, therefore, deserve further investigation.

## Graphical abstract

This article summarizes the state of knowledge regarding the effect of strain on cells involved in the fracture‐healing process.

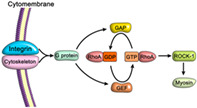

## Introduction

Fracture healing is a complex process that can be divided into the following stages: hematoma, granulation tissue formation, callus formation, and bone remodeling. The healing process is carried out by specific key cells including inflammatory cells, endothelial cells, fibroblasts, mesenchymal stem cells (MSCs), osteoblasts, and osteoclasts; not only the cells but also their secreted substances are important^1^, ^2^. Of note, the biological activities of these cells are regulated by mechanical stimuli. For instance, bone cells perceive mechanical stimulation (*via* receptor‐based interactions) and transduce the signal to osteoblasts or osteoclasts. In fact, mechanical stimulation regulates the process of osteogenic differentiation. Osteoblasts are mainly derived from MSCs; they can continue to differentiate into bone cells and are embedded in the bone mineralized matrix. Osteoclasts are derived from hematopoietic stem cells under the influence of mechanical factors; they differentiate from monocytes into multinucleated cells and perform the function of bone resorption. Importantly, different types of strain will have different effects on these cells^3^, thereby affecting fracture healing. In addition, proper relative motion between fracture fragments can stimulate bone callus formation and accelerate healing^4^, ^5^. Some researchers have proposed that by maintaining a 2%–10% strain between fracture ends, relatively stable secondary fracture healing can be achieved^6^, ^7^. Conversely, excessive or insufficient strain will affect this process and lead to non‐union or delayed union^8^, ^9^. Both experimental and clinical studies have confirmed this hypothesis^10^, ^11^, ^12^.

Goodship and Kenwright^13^ randomly divided 12 sheep into two groups, then performed osteotomy on the tibia of the sheep (with a gap of 3 mm) and fixed the sheep tibia *via* external skeletal fixation to establish a fracture model. Importantly, the healing process was compared between a control group in which rigid fixation was maintained, and an experimental group in which mechanical stimulation (axial micromovement) was applied daily. The authors found that the level of bone callus formation and the stiffness of the fracture site measured both *in vivo* and *in vitro* were higher in the experimental group than in the control group. Using the same model of tibial fracture, Kenwright *et al*.^14^ treated 85 severe tibial fractures with external skeletal fixation; patients were treated with highly rigid fixation. In both the control and experimental groups, the same fixation was used; however, axial micromovement was applied across the fracture site for 30 min per day to patients in the experimental group. Importantly, the authors observed that delayed union occurred in patients from the control group.

This article reviews the available literature on the effect of applied strain in the proliferation, differentiation, and activity of different types of cells involved in fracture healing. This review aims to deepen the understanding of the fracture healing process and provide a reference for appropriate fracture treatments.

## Hematoma Period

### *Effect of Mechanical Stimulation on Recruitment of* *MSCs*

MSCs migrate to the fracture site from the surrounding tissues and blood circulation^15^. Recruitment of a sufficient number of MSCs at the initial stage of a fracture is a prerequisite and constitutes the cytological basis for fracture healing^16^. Matrix metalloproteinases (MMPs) and tissue inhibitors of metalloproteinases (TIMPs) are expressed by MSCs and are closely related to the cells' migration ability^17^, ^18^. Ries *et al*.^19^ knocked out the genes encoding MMPs and TIMPs in MSCs and found that the ability of the stem cells to cross the human extracellular matrix was significantly reduced in a manner similar to that resulting from treatment with MMP inhibitors. Sweeney *et al*.^20^ found that application of a 5% strain significantly increased the expression level of MMP‐2 in stem cells, indicating that the synthesis of MMPs is also affected by the mechanical environment. Kasper *et al*.^21^ applied mechanical stimulation to MSCs, which resulted in elevated expression levels of MMP‐2, MMP‐9, and TIMP‐2. Moreover, mechanical loading promoted the migration of MSCs. In contrast, treatment with MMP‐2 inhibitors reduced stem cell migration, suggesting that mechanical stimulation promotes MSC migration by inducing MMP and TIMP expression.

### *Effect of the Mechanical Environment on Granulation Tissue Formation*

When a fracture occurs, activated platelets release a variety of products including fibronectin (FN), platelet‐derived growth factor (PDGF), and transforming growth factor‐β (TGF‐β). These substances trigger an inflammatory response, allowing inflammatory cells including fibroblasts and endothelial cells to enter the fracture gap and form new blood vessels and granulation tissue^22^. The formation of blood vessels is extremely important in fracture healing; lack of or insufficient angiogenesis can significantly delay fracture healing and affect the healing outcome^23^, ^24^. The morphological structure of bone‐bridging tissue is determined by regenerated blood vessels, and the formation of blood vessels is subject to the mechanical environment^22^. Fang *et al*.^25^ found that disruption of either the mechanical environment or endothelial cell proliferation leads to the blockage of angiogenesis and bone formation, thus preventing normal osteogenesis and resulting in fibrous non‐union of the fracture. These results indicate the interdependence between the mechanical environment, angiogenesis, and bone formation during osteogenesis, suggesting that the induction of pro‐angiogenic genes and an appropriate mechanical environment are necessary for the generation of new vascular systems during osteogenesis.

Blood vessel formation after a fracture can be induced by a variety of cytokines, among which vascular endothelial growth factor (VEGF) plays the most critical role^26^. Groothuis *et al*.^27^ collected fracture hematomas from healthy patients and cultivated them for 3 days in a bioreactor by providing a mechanical environment similar to that of early fracture healing in humans. Compared with the control group without mechanical stimulation, exposure to an appropriate biomechanical environment promoted the formation of endothelial cell tubes and allowed more angiogenesis regulators (VEGF and TGF‐β) to be released into the hematoma. Moreover, the hematoma matrix maintained a high pro‐angiogenic capacity within the first 24 h after stopping mechanical stimulation, and the capacity was offset by inhibition of vascular endothelial growth factor receptor 2 (VEGFR2).

Therefore, mechanical stimulation can enhance the angiogenesis of early hematomas, and VEGFR2 seems to play an important role in this process, which can persist even after removal of the mechanical stimulation.

## Callus Period

### *Osteogenic Differentiation of Fibroblasts*

The fibroblasts involved in fracture healing are mainly derived from the endosteum and periosteum^28^, ^29^. Fibroblasts can produce matrix components such as collagen, elastic fibers, mesh fibers, and glycoproteins. To our knowledge, there have been no reports focusing on the influence of mechanical stimulation on fibroblast osteogenic differentiation. However, fibroblasts have been shown to undergo osteogenic differentiation under the regulation of specific factors. Onishi *et al*.^30^ found that when a fracture occurs, the body can release bone morphogenetic proteins (BMPs), which induce fibroblasts at the fracture site to differentiate into osteoblasts and promote bone healing. Go *et al*.^31^ introduced the gene encoding BMP‐2 into fibroblasts, which significantly increased the activity of alkaline phosphatase (ALP) and the total calcium content compared with those of the control group without the gene. In addition, the expression levels of osteogenic marker genes, including genes encoding integrin‐binding sialoprotein (IBSP), runt‐related transcription factor 2 (Runx2), and osteoblast‐associated transcription factors (Osterix), also increased. These results indicated that BMP‐2 can promote the osteogenic differentiation of fibroblasts. Similarly, Chen *et al*.^32^ infected fibroblasts with viruses expressing BMP‐7, which resulted in significant enhancement of osteogenic‐related gene expression, calcium deposition, and ALP activity, indicating that BMP‐7 also enhances the osteogenic differentiation of fibroblasts.

Yamamoto *et al*.^33^ established a procedure to directly convert human fibroblasts into osteoblasts by transducing some defined factors and culturing the cells in an osteogenic medium. The transduction of the osteoblast‐specific transcription factors Runx2 and Osterix combined with octamer‐binding transcription factor 3/4 (Oct4) and L‐Myc led to the conversion of 80% of the fibroblasts into osteocalcin‐producing cells. Furthermore, treatment of RXOL factors (Runx2, Osterix, Oct4, and L‐Myc) induced the mRNA expression of osteocalcin, ALP, and Runx2 at levels comparable to those observed in normal osteoblasts and MSC‐derived osteoblasts (MSC‐OBs). In contrast, OL (Oct4 and L‐Myc)‐transduced cells expressed Runx2 at a low level and did not significantly express mRNA for the Osterix gene. These results suggest that human fibroblasts can be transformed directly into osteoblasts by introducing four transcription factor genes (RXOL). However, RXOL is not the minimum essential combination of factors required to achieve some degree of osteoblast‐like conversion of fibroblasts. A smaller number of factors such as Osterix and Oct4, and even Oct4 alone, is sufficient to induce bone matrix production by fibroblasts.

In addition, fibroblast growth factor (FGF) can promote the osteogenic differentiation of fibroblasts^34^. The above‐mentioned studies indirectly confirmed the osteogenic potential of fibroblasts in the process of fracture healing by exploring some of the biological factors involved in fibroblast osteogenic differentiation.

### *Effect of Strain on the Differentiation of* *MSCs*

Different mechanical environments have different effects on MSC differentiation. Chen and Jacobs^3^ and Steward and Kelly^35^ suggested that different types of mechanical stimulation, including pressure, tension, and fluid shear, act on different stem cell receptors through different mechanical transmission pathways, converting physical stimulation into different biochemical reactions. A variety of osteogenic and chondrogenic differentiation markers including BMP2, Runx2, Sox9, and osteocalcin (OC) participate in the process of differentiation^36^, ^37^, ^38^. Therefore, the expression levels of these factors can also be used as a direct reference to assess the differentiation level and direction of MSCs^39^.

Haasper *et al*.^40^ applied cyclic stretching with a 2% strain for 1 h per day for three consecutive days to MSCs, which resulted in a significant increase in the Runx2 expression level. When the strain was increased from 2% to 8%, the expression level of Runx2 increased even further. Jagodzinski *et al*.^41^ also applied cyclic stretching to MSCs and found that after 4 and 7 days of stimulation with 8% strain, the ALP expression level was significantly higher in the MSCs than in the cells of the unstretched control group. Moreover, the ALP level was significantly higher under 8% strain than that under 2% strain. Although the expression level of osteocalcin was generally lower, the trend was the same according to strain as that observed for ALP. Sumanasinghe *et al*.^42^ applied stress to MSCs with 10% and 12% strain and found significantly increased BMP‐2 gene expression after 1 and 2 weeks compared with that of the control group without mechanical stimulation. Several studies have reported similar results^40^, ^43^, ^44^. Koike *et al*.^45^ applied tensile stress to mouse‐derived MSCs and observed that 0.8% and 5% strain significantly increased ALP activity and Runx2 expression levels, whereas 10% and 15% strain resulted in a significant decrease in ALP activity and Runx2 expression. These results demonstrate that although low strain can promote osteogenic MSC differentiation, high strain may inhibit it.

Xie *et al*.^46^ applied mechanical stimulation to MSCs and demonstrated that the expression levels of chondrocyte biomarkers such as glycosaminoglycan (GAG) and Col2α1 in the 5% and 10% strain groups were significantly higher than those in the 15% and 20% strain groups and in the control group without mechanical stimulation. Similarly, Yang and Men^47^ applied cyclic tensile stress to mouse MSCs for 1, 3, 5, or 7 days. After 8 days, the mRNA expression levels of the chondrogenic differentiation markers *Sox9* and *Col2* showed a gradual upward trend with increasing time of mechanical stimulation, and both markers were significantly higher than those in the control group without mechanical stimulation. Angele *et al*.^48^ cultured MSCs in chondrogenic medium and applied stress stimulation at multiple time points (1–7 days). After 14 and 28 days, the content of proteoglycan, collagen, and other cartilage matrices in the medium was significantly higher than that in the control group without stress stimulation.

**Fig. 1:**
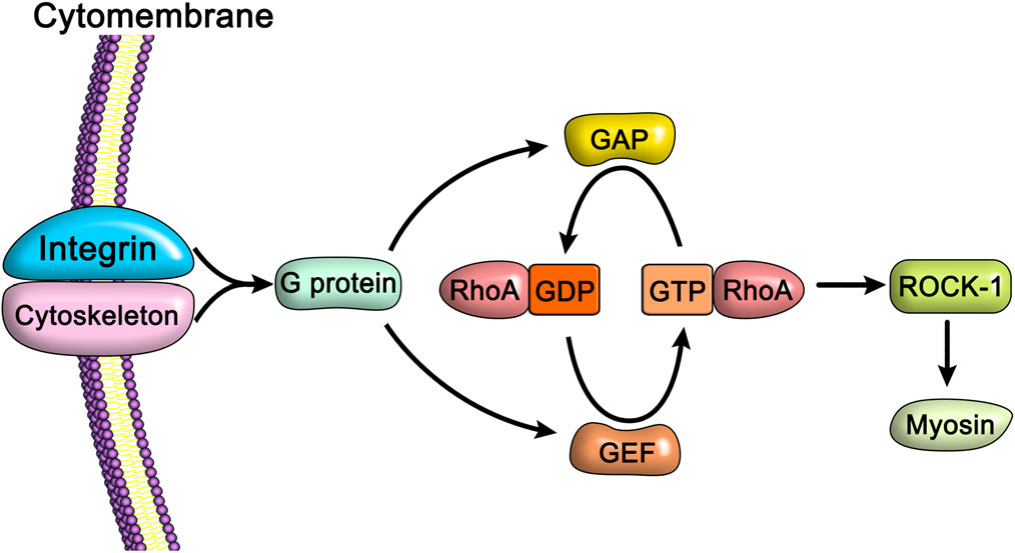
RhoA/ROCK‐1 signaling pathway of mesenchymal stem cells (MSCs). Transmembrane proteins, such as integrins, located on the MSC membrane can sense mechanical stimuli, convert them into mechanical signals, and subsequently transmit them to the G proteins that bind them. The G protein then transmits the signal into the cell to catalyze the transformation of RhoA to an active GTP state. The activation of RhoA triggers the downstream effector molecule ROCK‐1, which mediates the actions of myosin and determines the direction of MSC differentiation.

Collectively, these studies indicate that appropriate mechanical stimulation can promote chondrogenic MSC differentiation, which may be related to the RhoA/ROCK‐1 signaling pathway^49^, ^50^. Mechanical stimuli are sensed by transmembrane proteins (such as integrins) on the cell membrane. When integrin forms a complex with the cytoskeleton, the mechanical signal can be transmitted to the G protein bound to the complex, which then allows the G protein to transmit the signal to a series of other signaling pathways inside the cell, including the Rho/ROCK‐1 signaling pathway. RhoA exists in the form of binding guanosine diphosphate (GDP) and binding guanosine triphosphate (GTP), and plays a regulatory role through their mutual conversion. This process is mediated by guanine nucleotide exchange factor (GEF) and GTPase activating protein (GAP)^51^. The former catalyzes the transition of RhoA to an active GTP state, while the latter has an opposing effect and negatively regulates the activity of RhoA. The activation of RhoA triggers downstream effector molecule ROCK‐1, thereby affecting the role of myosin and determining the direction of MSC differentiation^52^ (Fig. 1).

### *Effect of Strain on the Proliferation and Differentiation of Osteoblasts*

Proliferation and differentiation of osteoblasts are regulated by various factors such as hormones, growth factors, and mechanical stimuli^53^, ^54^, ^55^, ^56^. Several factors are expressed during this process, including ALP, type I collagen fibers, and osteocalcin^55^, ^57^. Zhuang *et al*.^58^ applied mechanical stress to the MC3T3‐E1 osteoblast cell line with 0.17% strain and found that the number of cells was significantly higher in the cells under strain than that in the control group without strain. Similarly, Neidlinger‐Wilke *et al*.^59^ applied axial periodic stress to osteoblasts with strain of 1.0%, 2.4%, 5.3%, and 8.8%. Compared to that in the control group without mechanical stimulation, the proliferation of osteoblasts was significantly higher in the cells with strain below 1%; strain above 1% led to a reduction in cell proliferation. Kubota *et al*.^60^ and Ozawa *et al*.^61^ applied periodic and continuous mechanical stimulation to osteoblasts, respectively, and found that periodic mechanical loading increased osteoblast ALP activity and type I collagen content, while continuous mechanical stimulation showed opposite results. These studies demonstrate that only strain of appropriate size and frequency can promote the proliferation and differentiation of osteoblasts.

**Fig. 2:**
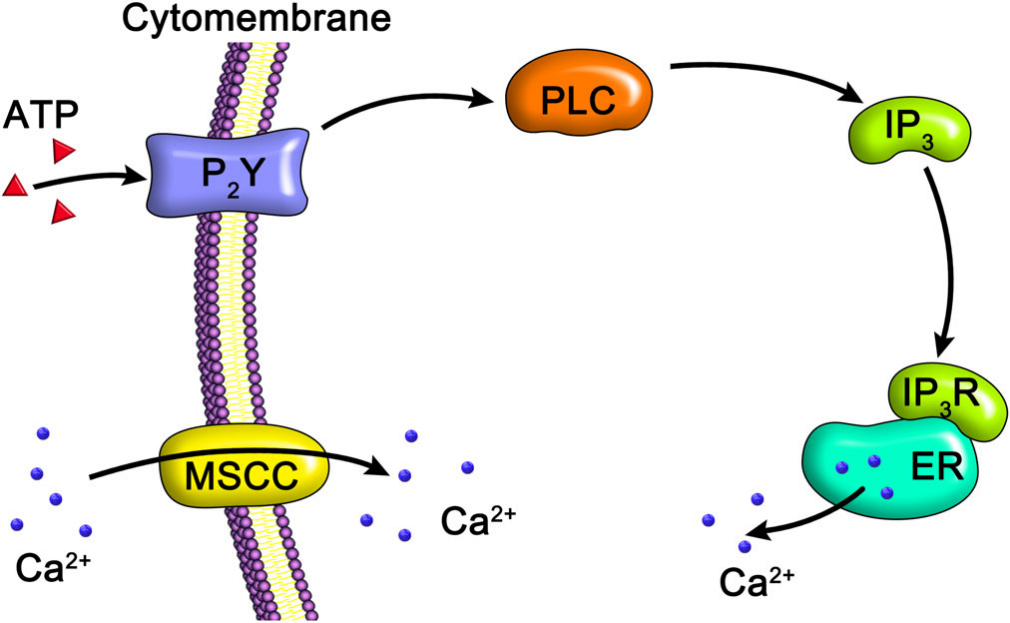
Mechanical stimulation‐induced calcium response mechanism of osteoblasts. The mechanical stimulation of osteoblasts causes the activation of mechanosensitive calcium channels (MSCC) on the membrane that allow the entry of extracellular calcium into the cytoplasm. Extracellular adenosine triphosphate (ATP) binds to the P2 purinoceptors on the cell membrane and triggers the G protein‐coupled receptor (GPCR) and phospholipase C (PLC) to produce inositol triphosphate (IP3). IP3 binds to its corresponding receptor on ER and releases calcium ions into the cytoplasm, thereby causing a series of reactions.

In addition, Weyts *et al*.^62^ applied periodic stress to osteoblasts at different time points, with strain ranging from 0.4% to 2.5%. When stress was applied on days 4–7 of culture, the total amount of cells decreased significantly and the number of apoptotic cells increased significantly. Conversely, when stress was applied on days 11–14 of culture, the total amount of cells increased significantly and the number of apoptotic cells did not change significantly. Moreover, when stress was applied on days 18–21 of culture, no significant change in the total amount of cells or the number of apoptotic cells was found. This indicates that osteoblasts have different responses to mechanical stimulation at different growth stages, which may be related to the different mechanical signal transduction mechanisms of cells at different growth stages^63^. Mechanical stimulation‐induced calcium response is the main regulatory mechanism of osteoblast differentiation. When osteoblasts are mechanically stimulated, extracellular calcium can enter the cytoplasm through mechanosensitive calcium channels (MSCC) on the membrane – the main source for the increase in intracellular calcium concentration – or the endoplasmic reticulum (ER). When osteoblasts are mechanically stimulated, extracellular adenosine triphosphate (ATP) binds to the P2 purinoceptors on the cell membrane^64^, activating the G protein‐coupled receptor (GPCR) and phospholipase C (PLC) to produce inositol triphosphate (IP3), which binds to the corresponding receptor on ER to release calcium ions into the cytoplasm and thus causing a series of reactions^65^ (Fig. 2).

## Remodeling Period

### *Effect of Strain on Osteoclasts*

**Fig. 3:**
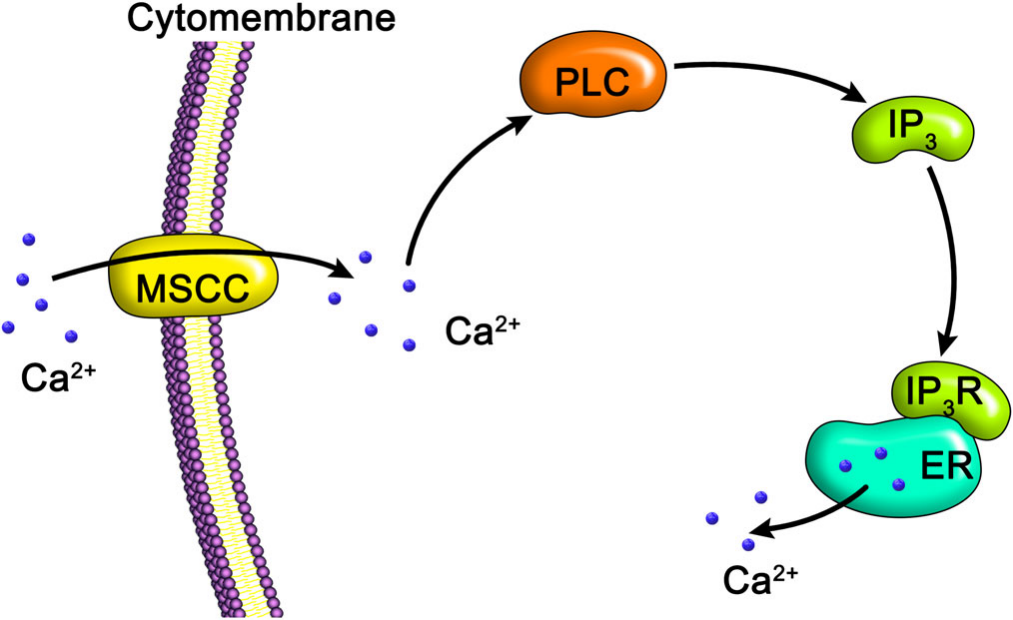
Mechanical stimulation‐induced calcium response mechanism of osteoclasts. When osteoclasts are mechanically stimulated, extracellular calcium can enter the cytoplasm through mechanosensitive calcium channels (MSCC) on the membrane and subsequently evoke phospholipase C (PLC) to produce inositol triphosphate (IP3). IP3 binds to its corresponding receptor on ER, thereby releasing calcium ions into the cytoplasm.

Osteoclasts are cells with bone matrix resorption function^66^. Mechanical stimulation is also involved in the regulation of osteoclasts^67^. Appropriate mechanical stimulation can inhibit osteoclast differentiation, interfere with their bone absorption function, and promote bone reconstruction^68^. This mechanism, similar to that of osteoblasts, mainly activates the MSCC on the cell surface, which then triggers the PLC in the cell, causing the release of calcium ions in the ER through the IP3 pathway^69^ (Fig. 3).

Studies have shown that the development and activity of osteoclasts are regulated by osteoblasts^70^. Osteoblasts express receptor activator of nuclear factor‐B ligand (RANKL), which interacts with the receptor activator of nuclear factor‐B (RANK) expressed on the surface of osteoclasts to promote their differentiation and activation^71^, ^72^. Moreover, osteoblasts also secrete osteoprotegerin (OPG), which is a decoy receptor for RANKL. OPG can occupy the binding site of RANK, thereby inhibiting the activation of osteoclast precursor cells^72^. When mechanical signals are input, the OPG/RANKL ratio can be changed to affect osteoclast activity^73^. Li *et al*.^74^ co‐cultured osteoblasts and osteoclasts and loaded them with a stress of 2500 με. The number of mature osteoclasts was then evaluated by counting tartrate‐resistant acid phosphatase‐positive polynuclear cells. The results showed that the stress culture medium significantly inhibited the differentiation of osteoclast precursor cells into mature osteoclasts. In addition, the OPG/RANKL ratio in osteoblasts under stress was significantly increased due to an increase in OPG levels. Moreover, by calculating the area of the bone resorption gap in the bone slice, the authors confirmed that mechanical stimulation significantly inhibited the bone resorption function of osteoclasts and reduced the formation of bone resorption lacunae. Xu *et al*.^75^ also found that low strain reduced the number of osteoclasts and the expression level of *RANK*, whereas high‐strain conditions produced the opposite result. Rubin *et al*.^76^ applied a 2% strain to murine bone marrow cells, which reduced osteoclast proliferation by 50%. This was attributed to the reduced expression level of osteoclast differentiation factor (ODF), as the expression of *Odf* mRNA in the experimental group was only 59% ± 3% that in the control group. In summary, proper mechanical stimulation can inhibit osteoclast differentiation and bone resorption.

## Conclusions and Future Prospects

In conclusion, recent studies have confirmed that most cells involved in fracture healing can respond directly or indirectly to mechanical stimulation and that the use of adequate mechanical stimulation is conducive to proper fracture healing. First, mechanical stimulation promotes the migration of MSCs and angiogenesis of early hematomas. Second, mechanical stimulation can promote the osteogenic differentiation of fibroblasts to aid the process of fracture healing. Third, adequate mechanical stimulation inhibits osteoclast differentiation and bone resorption, thus promoting bone reconstruction.

However, the effect of mechanical stimulation still depends on the loading method used. Thus, similar mechanical stimulations may cause different or even opposite cell responses. The reason may be that the same mechanical transduction pathway may share different mechanical stimuli and that different signal pathways may interact with each other^77^, ^78^. Furthermore, experimental studies on the response of cells to mechanical stimulation have mainly focused on a single type of cell. However, there are specific communication pathways and interactions among various cell types involved in bone healing, such as the above‐mentioned cooperation of osteoblasts and osteoclasts in bone reconstruction. As a result, there is still no unified conclusion as to how mechanical factors promote the differentiation of these cells. Therefore, elucidation of the mechanical signal transduction pathways and mechanical regulation between cells is a challenge that should constitute the focus of research in the future.

## Authorship Declaration

All authors listed meet the authorship criteria according to the latest guidelines of the International Committee of Medical Journal Editors, and all authors are in agreement with the manuscript.
